# Green Synthesis of Magnetic Nanoparticles Using Satureja hortensis Essential Oil toward Superior Antibacterial/Fungal and Anticancer Performance

**DOI:** 10.1155/2021/8822645

**Published:** 2021-01-19

**Authors:** Shahram Ahmadi, Mohammad Fazilati, Habibollah Nazem, Seyyed Mojtaba Mousavi

**Affiliations:** ^1^Department of Science, Payame Noor University (PNU), Tehran, Iran; ^2^Department of Chemical Engineering, National Taiwan University of Science and Technology, Taipei, Taiwan

## Abstract

The biological synthesis of nanoparticles, due to their environmental and biomedical properties, has been of particular interest to scientists and physicians. Here, iron nanoparticles (FeNPs) were synthesized using *Satureja hortensis* essential oil. Then, the chemical, functional, and morphological properties of these nanoparticles were characterized by typical experiments such as Uv-Vis, FTIR, XRD, FE-SEM, PSA, zeta potential, EDX, and EDX mapping. The results indicated Fe nanoparticles' formation with a cubic morphological structure and a particle size in the range of 9.3-27 nm. The antimicrobial effects of these nanoparticles were further evaluated using disc diffusion, minimum inhibitory concentration (MIC), minimum bactericidal concentration (MBC), and minimum fungal concentration (MFC) against two gram-positive bacterial strains (*Staphylococcus aureus* and *Corynebacterium glutamicum*), two gram-negative bacterial strains (*Pseudomonas aeruginosa* and *Escherichia coli*), and one fungus species *Candida albicans*. The results showed that green-synthesized Fe nanoparticles possessed higher antimicrobial properties than Satureja hortensis essential oil against selected pathogenic microorganisms, especially Gram-negative bacteria. Finally, the anticancer effect of these Fe nanoparticles was investigated on human cancer cells, K-562, and MCF-7, by the MTT assay. The results showed the anticancer effect of these nanoparticles against selected cell lines.

## 1. Introduction

**Figure 1:**
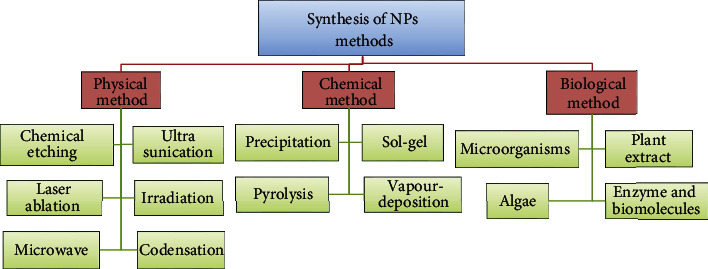
Different methods for the synthesis of nanoparticles.

The desire to produce and use of nanometer dimensions is increasing due to the interesting industrial characteristics of these materials and their wide range of applications in various fields of biology, drugs, and medicine [1–3]. A vast range of drugs, such as small hydrophobic and hydrophilic drugs, vaccines, and biological molecules, can be guided by these nanoparticles (NPs) [4, 5]. Nanoparticles are widely used to improve treatment and diagnose diseases [6–8]. Nanoparticles in nanoliposomes, carbon nanotubes, nanofibers, and nanocarriers have been widely used as drug carriers and cell scaffolding [9–11]. Nanoparticles enable the delivery of a significant type of drugs, including anticancer drugs, anti-Alzheimer's drugs, cardiovascular drugs, protease inhibitors, and several macromolecules into the brain cells [12]. On the other hand, diseases of microbial and fungal origin are among the most well-known diseases that have always plagued humans, and numerous studies have been conducted to identify, control, and treat these pathogens [13–15]. Bacteria strains, such as *Staphylococcus aureus*, *Escherichia coli*, *Streptococcus pyogenes*, and *Pseudomonas aeruginosa*, as well as *Candida albicans*, are among the pathogens that cause many problems in human's life [13–15]. Today, many effective antifungal and antibacterial drugs treat these infectious agents [16, 17]. However, due to the genetic diversity of microbial pathogens, the emergence of resistant strains, and the side effects of these drugs, the replacement of chemical drugs with antimicrobial drugs of living origins such as plants, algae, bacteria, and fungi is incredibly important and has led to research into the use of new drugs without side effects [16, 17]. The use of metal nanoparticles is one of these new drugs that has attracted particular attention and replaced chemical drugs and also do not have the effects mentioned above [18]. There are different ways to produce NPs, such as physical, chemical, and biological methods stated in Figure 1 [19, 20].

Nanoparticles resulting from the chemical methods used today have caused many worries, due to the use of hazardous and toxic chemical materials, as well as an environmental problem [21–25]. Among nanoparticles, silver, gold, and iron nanoparticles have received more attention due to their biological significance and medical applications. However, the chemical methods of preparing these NPs have left some toxic reactions that prevent the use of nanoparticles in biological applications [26–28]. The production of NPs using green chemistry has found a special place in research, and various types of biological systems are used for this purpose [29]. The use of microparticles, diatoms, and optical eukaryotes is one of these methods. But they are less commonly used due to their high cost of production and maintenance [30]. Today, plants and agricultural products have received particular attention as renewable sources for biomaterials [29, 31, 32]. Therefore, the identification of useful materials of native plants, their extraction for the production of nanoparticles, and their medical applications has become very important due to the problems mentioned above and the diversity of climates and plant flora in Iran [32]. These features are especially important for plants, such as *Satureja hortensis* (SH), which is exclusively native to Iran and has received little studies [31, 33–35]. Satureja hortensis is one of the plants from the mint family that has not only many applications in traditional medicine but also the antimicrobial activity of its phenolic compounds, thymol, and carvacrol has been proven to some extent on some fungal species [36–39]. In this research, therefore, green synthesis of FeNPs was performed using SH essential oil because of the mentioned problems and the importance of Satureja hortensis plant. Subsequently, the accuracy of the formation of synthesized nanoparticles, photochemical and morphological characteristics, particle size, active functional groups, and existing ions was investigated using conventional methods. The MIC, MBC, and MFC efficiencies of green synthesized FeNPs and SH essential oil were evaluated on five selected pathogenic microbes, including four bacteria and one fungus. Finally, the anticancer performances of the green-synthesized magnetite NPs were examined against K-562 and (Michigan Cancer Foundation) MCF-7 human cancer cells.

## 2. Materials and Methods

### 2.1. Materials and Collection of Satureja hortensis

Most of the materials, such as ferrous sulfate (FeSO4·7H2O), ferric chloride (FeCl3·6H2O), aqueous ammonia (NH3·H2O), ethanol (C2H5OH), and Muller-Hinton media, were purchased from Merck Company, Germany, and other purchased materials are listed elsewhere in the article. All glassware was washed with distilled water and sterilized before usage. *Satureja hortensis* plant was collected from Shiraz and Gachsaran (southwestern, Iran). Then, plant samples were transferred to the herbarium of the Faculty of Agricultural Sciences and Natural Resources, Shiraz University, for examination and confirmation, after which they were transferred to the laboratory for further experiments.

### 2.2. Preparations of the Plant Extract

Herein, the aerial part of the Satureja hortensis plant was first selected and thoroughly washed with deionized water to eliminate contaminants and then dried in the shade at the laboratory temperature of 25°C for seven days. Then, plant material was powdered by a grinder apparatus (IKA model A10 BASIC, Germany). The SH essential oil was extracted using a rotary evaporator according to the protocol provided by Mousavi et al. [39]. To this end, 10 g of Satureja hortensis powder was poured into a flask, and then 90 V% of ethanol was added to the flask to soak the powdered plant. The suspension containing Satureja hortensis essential oil was filtered after 24 hours. The remaining solution, which contained dissolved components of Satureja hortensis and ethanol, was kept at 1-3°C under suitable conditions for continued operation. Subsequently, an amount of 90 Vol% ethanol was added to the residual condensate and filtered after 24 hours. In order to increase the concentration and purity of the SH essential oil and also to altogether remove ethanol from the extract for antimicrobial testing, both solutions were combined and placed in a rotary evaporator. The production of Satureja hortensis essential oil continued until it reached a minimum concentration and the highest purity. Finally, the extract was stored in suitable conditions (1-3°C) for further testing.

### 2.3. Preparation of Green Fe_3_O_4_ Nanoparticles

Biosynthesis of FeNPs was performed using a simple multistep method according to the protocol stated by Mousavi et al. [39]. For this purpose, 3.89 g (14 mmol) of ferrous sulfate (FeSO4·7H2O) and 4.55 g (28 mmol) of ferric chloride (FeCl3·6H2O) were weighed and added to a glass jar containing 200 ml of concentrated Satureja hortensis essential oil. In order to obtain a homogeneous suspension, this solution was then stirred at 80°C for 30 min. Additionally, 30 ml of aqueous ammonia (NH3·H2O) (25%) was pipetted slowly to the obtained suspension and stirred for 1 h at 80°C, similar to the previous step. Next, the produced FeNPs were separated using a strong magnet, and the resulting sediment was washed three times by deionized water to neutralize the resulting magnetic NPs. Eventually, the FeNPs were dried for 1 h at 100°C.

### 2.4. Characterization of Fe Nanoparticles

Ultraviolet-visible (Uv-Vis) spectroscopy is the most important technique and the simplest method to confirm the formation of metal NPs. The absorbance spectrum of the colloidal sample was obtained in the range of 200–900 nm, using a UV–Vis spectrometer (ʎambda35, PERKIN Elmer, England) with distilled water reference. Fourier transform infrared (FTIR) spectroscopy was performed to classify the biomolecules in SH essential oil, which was responsible for the reduction of the iron metals and the stabilization of NPs. The functional group responsible for the FeNPs was also analyzed by FTIR (Bruker model Tensor II) in the wavelength range 4000–400 cm^−1^. The crystalline nature of green-synthesized FeNPs was confirmed by the X-ray diffraction (XRD) pattern. XRD data were recorded by XRD-7000 (Shimadzu, Japan) using monochromatic CuK*α* radiation (*λ* = 1.54056°A) operated at 40 kV and 30 mA at a 2*θ* angle pattern. The scanning was done in the region of 10^o^–80^o^. The images obtained were compared with the Joint Committee on Powder Diffraction Standards (JCPDS) library to account for the crystalline structure. The morphology and shape of the FeNPs were examined using field emission-scanning electron microscope (FESEM) (Tescan model Mira III) at a voltage of 200 kV. EDX and EDX mapping (Tescan model S Max detector Mira III) analysis were used to confirm the presence of elemental Fe in biosynthesized FeNPs. Additionally, zeta potential, polydispersity, and size distribution measurements of FeNPs were carried out by using the zeta potential (Horiba model SZ-100, Japan) and Particle Size Analyser (PSA) (Malvern model MS1002, England), respectively, after through sonication of synthesized NPs which were diluted by double-distilled water.

### 2.5. Evaluation of Antimicrobial Effects of Synthesized Materials

#### 2.5.1. Reference Strains and Preparation of Test Samples

In this study, five references of pathogenic microbial strains were selected, including two G^−^ bacteria (*Pseudomonas aeruginosa* (ATCC 10662) and *Escherichia coli* (ATCC 33876)), two G^+^ bacteria (*Staphylococcus aureus* (ATCC 6538) and *Corynebacterium glutamicum* (ATCC 21799)), and one fungal species, *Candida albicans* (ATCC 10231). Microbial samples were purchased from the Iranian Biological Resources Centre (IBRC). In order to evaluate the antimicrobial activity, microbial strains were grown in Muller-Hinton broth (MHB) medium for 24 h at 37°C, and after the incubation period, turbidity was adjusted to approximately 10^6^ CFUs/mL. Also, serial dilutions (from 7.8 to 100 *μ*g/ml) were prepared from the synthesized FeNPs by dissolving in 5% dimethyl sulfoxide (DMSO) for further experiments. MICs of the biosynthesized FeNPs were determined using broth microdilution methods.

#### 2.5.2. Antimicrobial Effects of Satureja hortensis Essential Oil and Green Synthesized FeNPs

Herein, the antibacterial and antifungal activities of FeNPs and SH essential oil against the five selected microbial strains were determined using the agar disc diffusion method. For this purpose, the selected pathogenic bacteria were cultured in MHB medium for 24 h, and then 100 *μ*l of this bacterial suspension was spread entirely on the surface of the cultivated plates containing the MHA medium in sterile conditions. Sterile paper discs of 6 mm in diameter were impregnated with 25 *μ*l of the tested compounds and placed on the plates' surface containing the cultured bacteria. Subsequently, these plates were incubated at 37°C for 24 h. The stages of the antifungal effects of the selected compounds in this experiment were the same as those of the antibacterial test, but the only difference was in the selected culture medium. Herein, potato dextrose broth (PDB) and potato dextrose agar (PDA) media, specific to fungal growth, were used, respectively, instead of MHB and MHA. In this experiment, amoxicillin and ketoconazole were used as a positive control for antibacterial and antifungal agents, respectively. The test was done three times to ensure its accuracy and precision. The average number obtained was considered as the zones of inhibition after incubation.

#### 2.5.3. Determination of MIC, MBC, and MFC Performance of Satureja hortensis Essential Oil and Green Synthesized FeNPs

In this section, the antimicrobial performance of FeNPs and SH essential oil to the five selected microorganisms was evaluated by MIC, MBC, and MFC assays. Similar to the previous step, the selected bacterial strains and the fungus were incubated in the MHB and PDB media, respectively, at 37°C for 24 h until obtaining turbidity of approximately 10^6^ CFUs/mL (0.5 McFarland). MIC of the synthesized FeNPs was determined using the microdilution assay. Then, 2-fold serial dilutions of green-synthesized FeNPs and SH essential oil were made in sterile MHB medium from a low concentration of 7.8-1000 *μ*g/mL. After that, 100 *μ*L of the FeNPs and SH essential oil dilutions was added to a 96-well microplate, inoculated with 100 *μ*L of the selected microbial strains to a final concentration of 5 × 10^5^ CFU/mL, and further incubated (with shaking) at 37°C for 24 h. The MIC of green-synthesized FeNPs was measured as the lowest concentration at which the growth of pathogenic microbial was completely inhibited within 24 h from incubation at 37°C. In this experiment, amoxicillin and ketoconazole were used as a positive control, and normal saline and a sample without FeNPs were used as two negative controls. The optical density was measured by a microplate reader (Hyperion, model MPR4 Plus) at 600 nm. In order to determination of MBC and MFC, 20 *μ*L of each bacterial strains and the fungal species was spread on MHA and PDA plate and further incubated at 37°C for 24 h. The plates with no bacterial and fungal growth were considered as the MBC and MFC, respectively.

### 2.6. Anticancer Performance of the Synthesized Compound

#### 2.6.1. Preparation of Cancer Cells and Cell Culture

To perform this experiment, the human breast cancer cells (MCF-7) and chronic myeloid leukemia (K-562) were obtained from the Cancer Cell Bank of Shiraz University of Medical Sciences and transferred to the Research Laboratory of Fars Science and Technology Park Company. Selected cell lines were cultured in RPMI1640 medium (Sigma Co, England) containing 10% fetal bovine serum (FBS), 2 g/L of bicarbonate, 2 mL of glutamine, and 100 mg/ml of penicillin-streptomycin. Then, it was incubated at 95% moisture with 5% carbon dioxide at 37°C, so that the number of cells reached 10^6^ cells.

#### 2.6.2. Cell Toxicity of FeNPs and Satureja hortensis Essential Oil Using the MTT Method

The efficacy of green-synthesized FeNPs and SH essential oil on the growth and proliferation of cancer cell lines was examined by the colorimetric method, methyl thiazol tetrazolium (MTT). Due to the mitochondrial activity of living cells, tetrazolium salts are reduced to Formosan crystals with different absorptions [40, 41]. To perform this method, 100 *μ*L of prepared cellular suspension was seeded in each cell of a 96-cell microplate, so that each mL of culture medium contained 10,000 cell lines. After 24 h of incubation, different concentrations (1, 10, 50, 100, 200, and 500 *μ*g/mL) of synthesized FeNPs and SH essential oil were incubated. After 24 h of treating cells with FeNPs, 20 *μ*L of MTT color (Sigma Co., Germany) with a concentration of 5 mg/ml was added to each microplate cell, and the plate was incubated in a CO_2_ incubator at 37°C in dark conditions for 2 h. After this time, the culture medium containing the MTT color was carefully taken out, 54 *μ*l of DMSO was added to each well of a microplate and shaken to dissolve the purple crystals Formosan. Finally, the optical absorption of each well, 40 min after incubation, was measured using a microplate reader (Hyperion, model MPR4 Plus) at a wavelength of 570 nm. The results were considered as IC50 in terms of cellular survival and efficacy, which inhibited cell growth by up to 50%. The experiments of this study were repeated three times, Busulfan (Myleran) was used as a positive control, and DMSO was applied as a negative control. The cell viability was computed using the following formula:

(1) $$\begin{array}{c} \text{Cell survival rate}=\left(\frac{\text{absorption of treated cell lines}}{\text{absorption of control cell lines}}\right)\times 100. \end{array}$$

### 2.7. Statistical Analysis

In this research, the results were evaluated based on mean ± standard deviation (SD) using SPSS software version 22 (SPSS, Inc., Chicago, IL, USA) by one-way ANOVA and Tukey's statistical tests. Values of *P* < 0.05 were considered statistically significant. All tests for the antimicrobial and anticancer effects were repeated three times. Graphs were plotted in Excel (2010).

## 3. Results and Discussion

### 3.1. Green Synthesis of FeNPs

Visual observations of the color change of synthesized FeNPs by SH essential oil from light yellow to burnt chocolate indicate the reduction of FeSO_4_ and the formation of FeNPs. This color change of FeNPs was due to the surface plasmon resonance (SPR) resulting from the formation of FeNPs [42]. The exact mechanism of green synthesis of FeNPs using the plant is set in the early step. However, researchers suggest that the enzymes and compounds found in plant extracts are an essential section of the green synthesis of NPs [43–45]. It is also believed that biomolecules, including phenol, protein, and flavonoids in SH essential oil, play an essential role in reducing nanoions as a coating or reducing NPs [46, 47].

### 3.2. Characterization of Synthesized FeNPs

#### 3.2.1. UV–Vis Spectrophotometry

**Figure 2:**
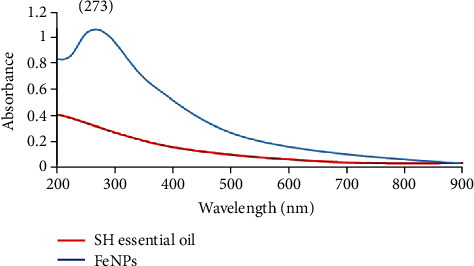
UV-Vis spectrum of synthesized Fe NPs using SH essential oil.

The bioreduction of FeNPs was further explained by measuring their absorbance with a UV-Vis spectrophotometer over the range of 200-900 nm. In this research, synthesized FeNPs had an absorption peak at 273 nm (Figure 2). These results are in harmony with Saranya et al. [47] and Pattanayak and Nayak [48], who reported the biosynthesis of FeNPs from *Musa ornat*e and *Azadirachta indica*, respectively, and showed sharp peaks in the range of 250-350 nm in UV–Vis spectra. Absorption peaks between 200 and 300 nm area were observed owing to the excitation of surface Plasmon vibrations in FeNP solution, which is identical to the characteristic UV-Vis spectra of Fe oxide NPs. On the other hand, the formation of FeNPs is known to occur through the complexation of Fe salts followed by capping of Fe with phenolic compounds [49–52].

#### 3.2.2. XRD Spectra of Green-Synthesized FeNPs

**Figure 3:**
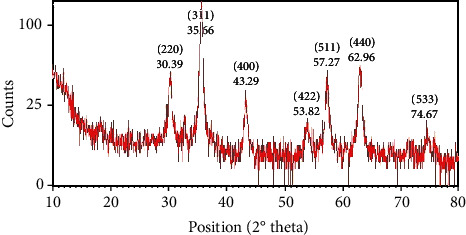
XRD patterns of synthesized FeNPs by Satureja hortensis for the determination of Fe crystals.

XRD patterns in Table 1 and Figure 3 demonstrate that the FeNPs are truly crystalline and display diffraction peaks at 2*θ* values of 30.2944, 35.668, 43.2994, 53.8234, 57.2722, 62.9664, and 74.6793, which are corresponding to amorphous structure (220), (311), (400), (422), (511, 440), and (533) planes of cubic crystal system of iron oxide (Fe_3_O_4_). The position and relative intensity of diffraction peaks are the same as the standard data for bulk magnetite (JCPDS file No. 96-900-5842), indicating the purity of synthesized FeNPs. Our XRD results support those reported previously [36, 53–55].

#### 3.2.3. FTIR Profile of Green-Synthesized FeNPs

**Figure 4:**
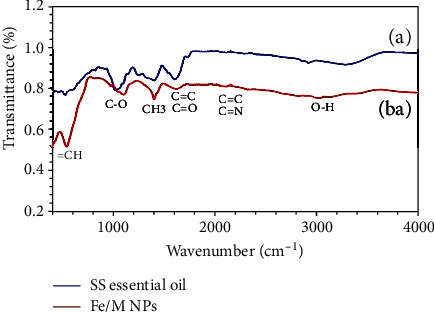
FTIR spectrum of (a) Satureja hortensis essential oil and (b) synthesized Fe NPs.

FTIR assay was applied to recognize the biomolecules that may be responsible for the reduction of metal ions and stabilization factors for NPs. An FTIR spectrum of SH essential oil formed in a limited 400-4000 cm^−1^ as shown in Table 2 and Figure 4. A band observed at about 3026.74 cm^−1^ of FTIR in the sample is related to the O-H group from alcohols/phenols [56, 57], or N-H is stretching of amide A [57, 58], suggesting that this stretch could be responsible for the reduction of FeSO_4_. Another band at 2112.97 cm^−1^ FTIR spectrums is indicative of C ≡ C, and C ≡ N stretches of aliphatic/aromatic compounds [55, 59]. Also, the bands from 1621.78 cm^−1^ to 1767.10 cm^−1^ demonstrate the C=C group from aromatic compounds [55, 57, 60]. A previous report determined these band signs for amid I (C=O stretch) protein [58]. The broad peak of about 1402.83 cm^−1^ was attributed to methyl C-H stretching from carbohydrates [57, 61]. The presence of a band at 1103.25 cm^−1^ to 977.71 cm^−1^ is related to the C-O stretch in-plane bending of alkanes, alcohols, carboxylic acids, esters, and ethers. Eventually, a band observed about 543.61 cm^−1^ could represent = CH group in aromatic bicyclic monoterpenes, demonstrating the Fe_3_O_4_-NPs [53, 62–64]. Identified functional groups are found in previous FTIR analysis of Fe_3_O_4_-NPs synthesized by Tie Guany in tea extract [62] and aqueous extracts of *Sageretia thea* [65]. The comparison of derived results from this study with those presented on the synthesis of FeNPs indicates the accuracy of the synthesized NPs.

#### 3.2.4. EDX and EDX Mapping Analysis

**Figure 5:**
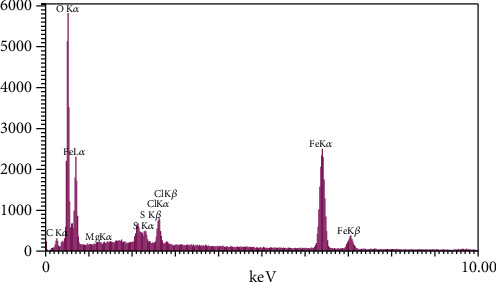
EDX spectrum of green-synthesized FeNPs.

The EDX spectroscopy analysis of synthesized FeNPs using SH essential oil is shown in Figure 5, indicating the collected nanoparticles' elemental composition. These EDX data are beneficial in reflecting the nuclear content on the surface area of the FeNPs. The EDX spectrum showed strong peak signals of Fe with K*α*, K*β*, and L*α* peaks at 6.4, 7.0, and 0.75 keV, respectively. EDX quantification gives atomic percentages of 19.61%, 67.37%, 10.23%, 1.07%, 1.30%, and 0.42% for C, O, Fe, S, Cl, and Mg, respectively (Table 3). The presence of Fe and O illustrates that the NPs exist in oxide form, Fe_3_O_4_ [50, 66]. The presence of C and O ions in the EDX spectrum was related to polyphenols or each compound containing C and O in SH essential oil [66, 67]. The S and Cl peaks signify the presence of sulfate and chlorine groups of the applied FeSO4 and FeCl3 precursors, respectively. The existence of Mg ion in the EDX spectrum is related to the complex of SH plant extract. Probably magnesium plays an important role in enzyme activity. Very similar outcomes of EDX were stated for FeNPs derived from other plant extracts [66, 68, 69].

**Figure 6:**
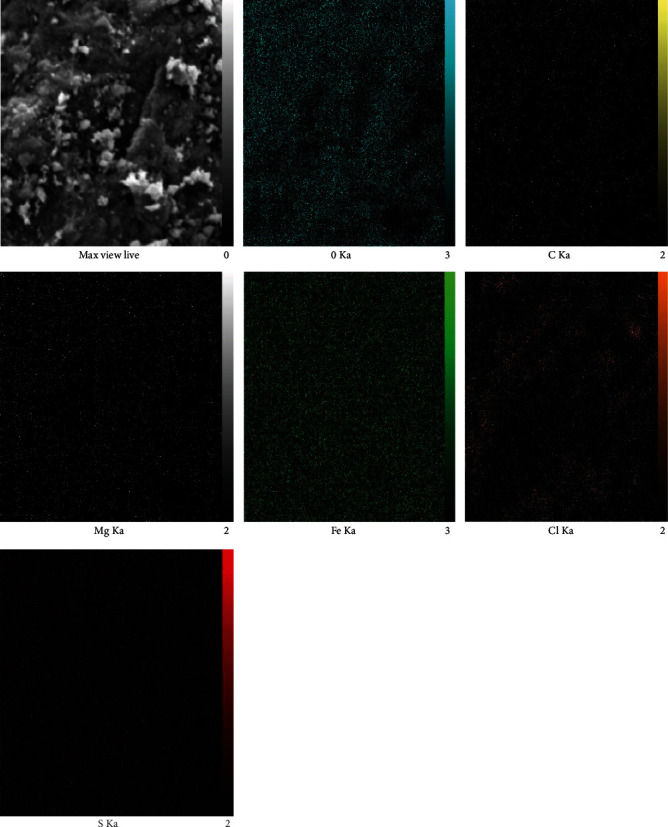
EDAX mapping analysis of green synthesized Fe nanoparticles.

The results obtained from biosynthesized FeNPs using the SH extract by the EDX mapping method are shown in Figure 6. Accordingly, the significant compositions of SH essential oil are truly distributed all over the FeNPs, as O and Fe are the essential elements of these NPs. Herein, the high quantity of Fe ions within these NPs emphasizes the successful synthesis of magnetite NPs with SH essential oil [70].

#### 3.2.5. FESEM Scanning

**Figure 7:**
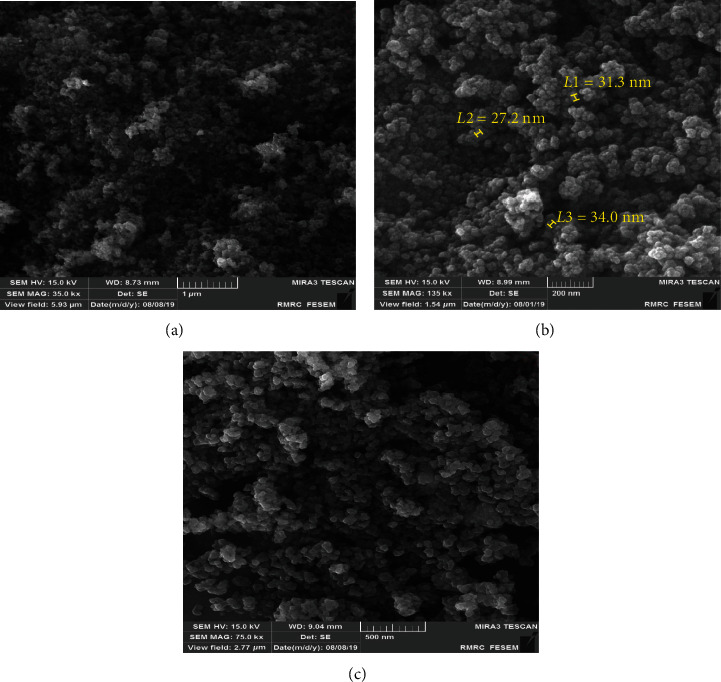
FESEM images of biosynthesized FeNPs using SH essential oil.

The formation of Fe_3_O_4_-NPs and its morphological dimensions was studied using the FESEM (Figure 7). The study demonstrated that the average size of the NPs was in a limited area of 27-34 nm. It also exhibits the formation of cube-shaped FeNPs. The formation of cube-shaped NPs was induced by enzymes, chlorine, and magnesium compounds present in the plant sample, which influences the nanoparticles' morphology. Because of a very narrow electron beam, FESEM micrographs have a complete depth of field that is a 3D appearance trait to understand a helpful sample [61, 71].

#### 3.2.6. Particle Size Analysis (PSA) and Zeta Potential Measurements

**Figure 8:**
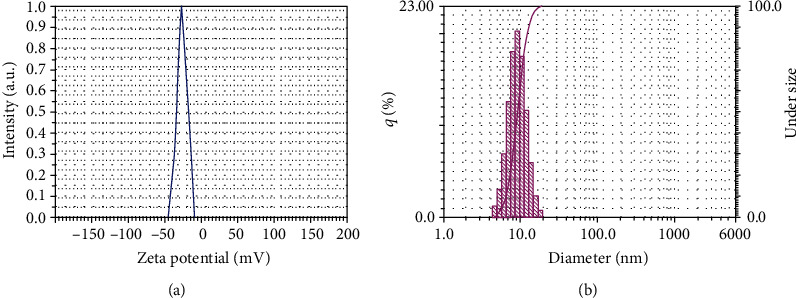
(a) The zeta potential of FeNPs synthesized by Satureja hortensis. (b) The PSA graph of synthesized Fe_3_O_4_ NPs using SH essential oil.

The surface potential of FeNPs was detected using the zeta potential method, which is a fundamental characterization tool to distinguish the stability of NPs in an aqueous solution. As depicted in Figure 8(a), the zeta potential of Fe_3_O_4_-NPs is -35 mV (at a range of -10 to -45 mV), demonstrating that the synthesized NPs are highly stable due to their strong negative surface charge [61]. The distribution size of the synthesized FeNPs was determined by PSA. As shown in Figure 8(b), an average size of 9.2 nm was obtained for the NPs synthesized in this experiment. The crystalline size of FeNPs calculated from PSA was further counterverified using XRD and FE-SEM methods.

### 3.3. FeNP Antimicrobial Performance

**Figure 9:**
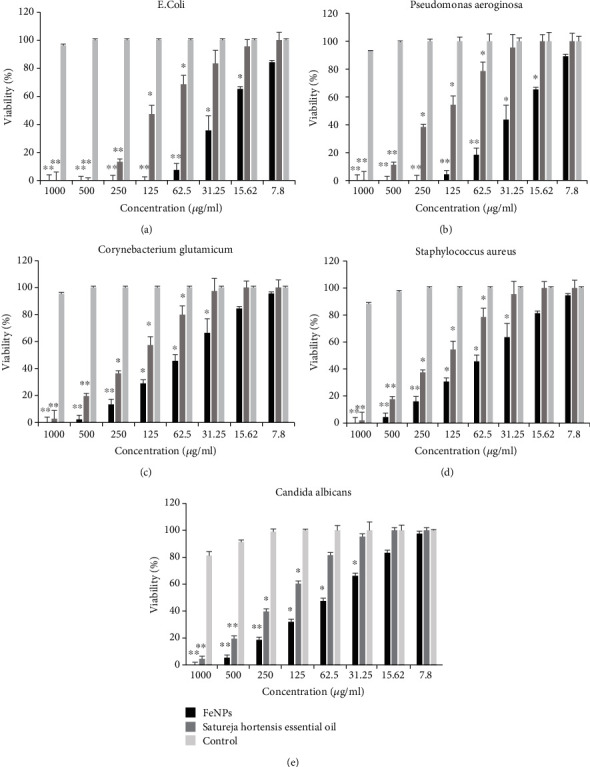
Comparative MIC data (*μ*g/ml) from different concentrations of FeNPs and SH essential oil against five pathogenic microbe strains. (a) *Escherichia coli*, (b) *Pseudomonas aeruginosa*, (c) *Corynebacterium glutamicum*, (d) *Staphylococcus aureus*, and (e) *Candida albicans.* Each bar represents the mean ± SD (standard deviation) of three independent tests; ^∗^ and ^∗∗^ represent statistical significance between control versus each microbe at ^∗^*P* < 0.01 considered significant and ^∗∗^*P* < 0.001 highly significant levels using (ANOVA)/Tukey tests.

In this section, the antimicrobial effect of different concentrations (7.8, 15.62, 31.25, 62.5, 125, 250, 500, and 1000 *μ*g/mL) of green-synthesized FeNPs and SH essential oil was examined against five microbial strains (*Staphylococcus aureus*, *Corynebacterium glutamicum*, *Pseudomonas aeruginosa*, *Escherichia coli*, and *Candida albicans*) using the disc diffusion method, followed by microdilution methods for obtained MIC, MBC, and MFC. The comparison of the antimicrobial effects of these synthesized NPs with SH essential oil and also with amoxicillin and ketoconazole as positive control samples was indicative of a high potential of the synthesized FeNPs against the selected microbes. The disc diffusion method (Table 4) showed that the highest diameter of antimicrobial inhibition zone was for Gram-negative bacteria, *E. coli*, and *P. aeruginosa* with 16.7 mm and 14.3 mm, respectively. The antimicrobial effect of the SH essential oil in this method was similar to that of synthesized FeNPs, except that the diameter of the inhibition zone was reduced (10.7 and 10.4 mm). As can be seen from Figure 9 and Table 5, in microdilution experiments, antimicrobial effects increased with the increasing concentration of synthesized NPs. High concentrations (500 and 1000 *μ*g/mL) of both FeNPs and SH essential oil had antimicrobial effects against five selected microbes. However, the effects of synthesized FeNPs were more significant at high and low concentrations. Similar to the disc diffusion test, MICs and MBC belonged to two Gram-negative bacteria strains, *E. coli* and *P. aeruginosa*, with concentrations of 31.25 *μ*g/mL and 62.5 *μ*g/mL, and 62.5 *μ*g/mL and 125 *μ*g/mL, respectively. As shown from the figure and table, SH essential oil in this method has similar results to the synthesized FeNPs. The MIC and MBC belonged to two gram-negative bacteria, *E. coli* and *P. aeruginosa*, with a concentration of 250 *μ*g/ml for each bacterial strain.

Regarding the antimicrobial effect of synthesized FeNPs against selected gram-positive bacteria strains and fungi species, the results were almost similar in both methods. In the disc diffusion method, the average diameter of the antimicrobial inhibition zone for selected microbes, *S. aureus*, *C. glutamicum*, and *Candida albicans*, was 12.25 mm. Furthermore, MIC in the microdilution method was at concentrations of 125, 250, and 250 *μ*g/ml, respectively. Whereas for SH essential oil, the inhibition zone's average diameter in the disc diffusion method was 10 mm and MIC in the microdilution method for all three microbes at a concentration of 500 *μ*g/ml. Notably, amoxicillin and ketoconazole as a positive control sample showed no antimicrobial properties compared to synthesized compounds. The antimicrobial activity of synthesized FeNPs was more because of smaller particles having a bigger surface area, which can enhance the capability to go through the cell membrane and provide more bactericidal effect. The Fe ions from FeNPs are suggested to become attached to the negatively charged bacterial cell wall and rupture it, which leads to denaturation of protein and finally cell death. Nanoparticles have a large surface-to-volume ratio, so it can firmly adhere to the cell surface of the fungus. Due to its small size, it can directly penetrate the cell and damage the cell wall. Inactivation of fungus by FeNPs involves the direct interaction between nanoparticles and cell surfaces, which affects the permeability of membranes where NPs enter and induce oxidative stress in fungus cells, subsequently resulting in the inhibition of cell growth and eventually cell death. Possibilities of membrane damage caused by direct or electrostatic interaction between FeNPs and cell surfaces, cellular internalization of NPs, and the production of active oxygen species such as H2O2 in cells due to metal oxides have been reported in the literature. A comparison of the present test results with those of previous researches on synthesized FeNPs using another plant extract suggested that the synthesized FeNPs had a high antimicrobial effect and could be used as an antimicrobial drug in medicine [39, 52–54, 66, 72].

### 3.4. Anticancer Effects of SH Essential Oil and Synthesized FeNPs against MCF-7 and K-562 Cell Lines

**Figure 10:**
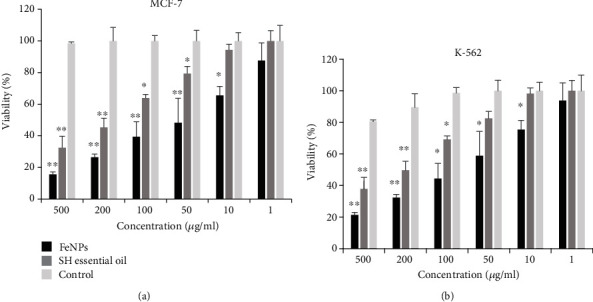
MTT colorimetric assay of cancer cell-cytotoxicity of synthesized FeNPs and SH essential oil on the MCF-7and K-562 human cancer cells at different concentrations. (a) MCF-7 cancer cell line, (b) K-562 cancer cell line. Each bar represents the mean ± SD (standard deviation) of three independent tests; ^∗^^∗∗^ represent statistical significance between control versus each MCF-7 and K-562 cancer cell lines at ^∗^*P* < 0.01 considered significant and ^∗∗^*P* < 0.001 highly significant levels using (ANOVA)/Tukey tests.

The expansion of cancer and its mortality has increased worldwide in recent decades [72, 73]. Moreover, the side effects of therapeutic drugs and the high expense of treatment have drawn the attention of researchers and pharmacists to the production of drugs without side effects using low-cost methods. Meanwhile, the synthesis of metal NPs using plants and herbal extracts as an anticancer drug without side effects and low production cost has attracted researchers and physicians [74, 75]. In this study, different concentrations (1, 10, 50, 100, 200, and 500 *μ*g/mL) of the synthesized FeNPs and SH essential oil were investigated for their anticancer performance against MCF-7 and K562 human cancer cell lines by the MTT colorimetric method. As shown in Figure 10, the synthesized FeNPs using SH essential oil had a higher antioxidant property than the SH essential oil. As can be deduced from the figure, both compounds had a carcinogenic effect at high concentrations of 200 and 500 *μ*g/mL compared to the control group. At low concentrations, however, the results were slightly different. At concentrations below 200 *μ*g/mL of the SH essential oil, the cell viability percent was reduced for both cell lines. It can be stated that the antioxidant property of the SH essential oil was reduced, or it had no anticancer activity against selected cancer cell lines. The anticancer effect of SH essential oil can be considered, due to the presence of phenolic acids and flavonoid compounds such as thymol, carvacrol, *ρ*-cymene, and *γ*-terpinene. Furthermore, these compounds have anticancer and antimicrobial properties [31, 32]. The anticancer mechanism of SH essential oil has not been determined yet. However, in some references, delay or inhibition of oxidative damage caused by free radical and nonfree radical species has been cited as a reason for these properties [32].

For synthesized FeNPs, on the other hand, low concentrations up to 50 and 100 *μ*g/mL for MCF-7 and K-562 cancer cells, respectively, reduced cell viability to 50%, suggesting that synthesized NPs had an anticancer effect in these concentrations. Therefore, it can be stated that the inhibitory concentration (IC50) value for synthesized FeNPs against MCF-7 and K-562 cancer cell lines was 50 and 100 *μ*g/ml, respectively. Moreover, at these concentrations, the synthesized NPs have a potential anticancer effect against selected cancer cell lines. However, at concentrations lower than the IC50 value, the antiproliferative effect of the synthesized NPs was significantly reduced. So that, at concentrations of 10 and 1 *μ*g/ml, synthesized FeNPs had no anticancer properties against both cancer cell lines. Finally, FeNPs synthesized by SH essential oil have potential antioxidant properties for the studied cell lines, especially MCF7, with an IC50 value of 50 *μ*g/ml, and in the future, it can be used as a potential anticancer drug to treat breast cancer cells and chronic myeloid leukemia. The results of this study were compared with previous reports on cancer cells using FeNPs [76–78].

Also, the exact mechanism of anticancer effects of FeNPs is yet unknown. Nevertheless, different hypotheses have been proposed by researchers in this regard, such as induction of apoptosis and cell cycle arrest through the mitochondrial pathway in some cancer cell lines [70, 79]. In some other references, the cytotoxicity of FeNPs is related to their size and shape. They believe that the smaller size of nanoparticles, as well as their cubic and spherical shapes, has potential anticancer effects. The most probable reason for this is that the iron nanoparticles can directly contact the cell surfaces and initiate cytotoxicity [80].

## 4. Conclusions

In this research, a relatively different and straightforward method for the first time was used for the synthesis of green iron magnetic NPs using the essential oil of the Satureja hortensis plant. Subsequently, the synthesized FeNPs were characterized using conventional methods such as Uv-Vis, FT-IR, XRD, FE-SEM, EDX, EDX mapping, PSA, and zeta potential. The evaluation results of synthesized NPs confirmed the existence of FeNPs with a cubic crystal structure and a size of about 9.2-27 nm. Then, the antibacterial effect of the FeNPs was investigated on five microbial strains, including two G^+^ bacteria, two G^−^ bacteria, and a fungal species. The results of this experiment demonstrate the antimicrobial activity of these NPs on selected microbes, especially Gram-negative bacteria, *E. Coli*, and *P. aeruginosa*. Finally, the anticancer effect of the synthesized FeNPs was investigated on the human cancer cells, MCF-7, and K-562, and the results showed the potential effect of these NPs on the target cell lines, especially MCF-7. Therefore, it can be concluded that synthesized Fe_3_O_4_ NPs in a new way can eliminate the disadvantages of physical and chemical methods and be used as a potential nanomedicine drug to treat microbial infections and control cancer diseases.

## Figures and Tables

**Table 1:** Output data of XRD assay from synthesized FeNPs using SH essential oil.

No peak	Pos. [°2Th.]	Pos. [°2Th.] of ref.	Plane (hkl)	Crystalline structure	Chemical formula	Crystallite size only [Å]	Microstrain only [%]	Ref.
1	30.2944	30.273	220	Cubic	Fe24.00O32.00	190.1562	0.775783	96-900-5842
2	35.668	35.656	311	Cubic	Fe24.00O32.00	326.295	0.385734	96-900-5842
3	43.2994	43.34	400	Cubic	Fe24.00O32.00	164.1367	0.636558	96-900-5842
4	53.8234	53.775	422	Cubic	Fe24.00O32.00	85.17223	0.999908	96-900-5842
5	57.2722	57.331	511	Cubic	Fe24.00O32.00	262.8147	0.306042	96-900-5842
6	62.9664	62.966	440	Cubic	Fe24.00O32.00	178.9751	0.4124	96-900-5842
7	74.6793	74.511	533	Cubic	Fe24.00O32.00	57.26613	1.109769	96-900-5842

**Table 2:** FTIR analysis of synthesized FeNPs using SH essential oil.

The peak for synthesized Ag NPs (cm^−1^)	Functional group	Compositions present
3026.74	O-H stretch N-H stretch	Phenols, alcohols Amide A
2112.97	C ≡ N stretch C ≡ C stretch	Aromatic or aliphatic
1767.10 to 1621.78	C=C stretch C=O stretch	Aromatic Amid I
1402.83	Methyl C-H group	Carbohydrates
1103.25 to 977.71	C-O stretch	Alkanes, alcohols, carboxylic acids, esters, and ethers
543.61	= CH stretch	Aromatic bicyclic monoterpenes

**Table 3:** Outcome of EDX assay of biosynthesized FeNPs and Satureja hortensis essential oil.

Compound	Element	Intensity	Weight %	Atomic %
Satureja hortensis essential oil	C	46.9	18.79	25.60
	O	419.0	58.17	59.49
	Na	307.2	13.02	9.26
	Mg	128.5	4.20	2.82
	Al	76.1	1.92	1.16
	P	22.1	0.44	0.23
	K	20.1	0.43	0.18
	Ca	133.4	3.04	1.24
Synthesized Fe NPs using SH essential oil	C	26.3	11.92	19.61
	O	510.8	54.56	67.37
	Mg	13.3	0.52	0.42
	S	87.7	1.74	1.07
	Cl	110.6	2.33	1.30
	Fe	605.1	28.93	10.23
	C	26.3	11.92	19.61
	O	510.8	54.56	67.37

**Table 4:** Average of the inhibition zone of FeNPs and Satureja hortensis essential oil against selected microbes.

Microorganisms	The average diameter of inhibition		
	Satureja hortensis essential oil	FeNPs	Control
*Pseudomonas aeruginosa* (ATCC 10662)	10.4 ± 1.2	14.3 ± 0.8	6.5 ± 0.4
*Escherichia coli* (ATCC 33876)	10.7 ± 0.5	16.7 ± 0.6	6.7 ± 0.1
*Staphylococcus aureus* (ATCC 6538)	9.2 ± 0.4	12.2 ± 1.2	6.4 ± 0.6
*Corynebacterium glutamicum* (ATCC 21799)	10.0 ± 0.2	12.5 ± 0.6	6.4 ± 0.5
*Candida albicans* (ATCC 10231)	9.8 ± 0.9	12.10 ± 0.4	7.4 ± 0.2

**Table 5:** Comparison of MIC and MBC/MFC data from SH essential oil and green synthesized FeNPs.

Microorganisms	SH essential oil (*μ*gmL^−1^)		FeNPs (*μ*gmL^−1^)	
	MIC	MBC/MFC	MIC	MBC/MFC
*Pseudomonas aeruginosa* (ATCC 10662)	250	250	62.5	125
*Escherichia coli* (ATCC 33876)	250	<250	31.25	62.5
*Staphylococcus aureus* (ATCC 6538)	500	500	125	250
*Corynebacterium glutamicum* (ATCC 21799)	500	<500	250	<250
*Candida albicans* (ATCC 10231)	500	500	250	<250

## Data Availability

The data were obtained through practical experiments on microbial specimens and cancer cells and are presented in the article and, if necessary, can be made available to the journal.
